# Length of stay and cost of care associated with admissions for atrial fibrillation among patients with cancer

**DOI:** 10.1186/s12872-022-02697-4

**Published:** 2022-06-17

**Authors:** Avirup Guha, Anubhav Jain, Ankita Aggarwal, Amit K. Dey, Sourbha Dani, Sarju Ganatra, Francis E. Marchlinski, Daniel Addison, Michael G. Fradley

**Affiliations:** 1 Harrington Heart and Vascular Institute, Case Western Reserve University, Cleveland, OH USA; 2 Cardio-Oncology Program, Division of Cardiology, The Ohio State University Medical Center, Columbus, OH USA; 3 Department of Internal Medicine, Wayne State University School of Medicine, Ascension Providence Rochester Hospital, Rochester, MI USA; 4 National Heart, Lung and Blood Institute, Bethesda, MD USA; 5 Cardio-Oncology Program, Division of Cardiovascular Medicine, Department of Medicine Landsman Heart and Vascular Center, Lahey Hospital and Medical Center, Burlington, MA USA; 6 Section of Cardiac Electrophysiology, Division of Cardiovascular Medicine, Perelman School of Medicine, University of Pennsylvania, Philadelphia, PA USA; 7 Cancer Control Program, Department of Medicine, Ohio State University Comprehensive Cancer Center, Columbus, OH USA; 8 Cardio-Oncology Program, Division of Cardiovascular Medicine, Cardio-Oncology Center of Excellence, Perelman School of Medicine, University of Pennsylvania, 3400 Civic Center Blvd, Philadelphia, PA 19104 USA

**Keywords:** Atrial fibrillation, Cardio-oncology, Cardioversion, Cancer

## Abstract

**Background:**

The aim of this study is to assess the burden of AF-related hospitalizations inclusive of inflation-adjusted cost-of-care and length-of-stay (LOS) among cancer patients and the impact of direct current cardioversion (DCCV) on these outcomes.

**Methods:**

Using the National Inpatient Sample (NIS), patients hospitalized with either a primary or secondary diagnosis of AF and comorbid cancer were identified and both cost of hospitalization and LOS were evaluated for each group. Subgroup analyses were performed for specific cancer types (breast, lung, colon, prostate and lymphoma), and those receiving DCCV.

**Results:**

The prevalence of co-morbid AF was 8.2 million (16%) and 35.5 million (10%) among those with vs. those without cancer, respectively (odds ratio = 1.6, 95% confidence interval = 1.5–1.7; *P* < 0.001). Over time, both primary and prevalent AF admissions among those with comorbid cancer increased from 1.1% and 12.3% in 2003 to 1.5% and 21% in 2015, respectively. The total cost of hospitalization increased 94.4% among those with AF and comorbid cancer compared to 23.9% among those without cancer. Among the subgroup of patients with comorbid cancer and primary admission for AF undergoing DCCV, length of stay (2.7 vs. 2.2 days; *P* < 0.001, model 1) and cost of care ($7,093 vs. 6,152; *P* < 0.001) were both significantly higher.

**Conclusions:**

AF related admissions are increasing for all populations especially amongst those patients with a comorbid diagnosis of cancer, including all cancer subtypes evaluated. Among those patients who underwent DCCV, cancer patients had longer length of stay and increased health care costs.

**Supplementary Information:**

The online version contains supplementary material available at 10.1186/s12872-022-02697-4.

## Background

Cardiovascular (CV) disease and cancer represent two of the largest global contributors to mortality [1]. Among CV disorders, the burden of arrhythmias, particularly atrial fibrillation (AF) continues to increase worldwide. Over the last 2 decades, the prevalence of AF has doubled and is currently estimated at 59.7 million, now affecting over 10% of octogenarians [2, 3]. AF is also particularly common in cancer patients—a study of 24,125 patients estimated the prevalence of AF to be 2.4% at the time of cancer diagnosis [4]. Additionally, surgical or medical therapies for cancer may have either direct or indirect effects on the development of arrhythmias frequently resulting in hospitalization for the management of these issues [5, 6].

Due to the increasing burden of disease, AF is associated with increased health care utilization and cost in the general population [7]. Inpatient management of AF in cancer patients can be especially challenging, and certain treatment modalities including anticoagulation, direct current cardioversion (DCCV) or ablation, may not be considered due to safety concerns or even implicit biases of the practitioners [8–10]. As such, we sought to assess the burden of AF-related hospitalizations inclusive of inflation-adjusted cost-of-care and length-of-stay (LOS) among cancer patients and the utilization of DCCV for the treatment of AF in these patients.

## Methods

The National Inpatient Sample (NIS), an inpatient database in the United States (US) developed as a part of the Healthcare Cost and Utilization Project (HCUP) from January 1, 2003, through September 30, 2015, was used for this study. NIS is an administrative database that captures a 20% stratified sample of US hospitals. Description of NIS is presented in supplemental methods. The data underlying this article were provided by HCUP under license. Data will be shared on request to the corresponding author with the permission of HCUP.

We identified all hospitalized adults (≥ 18 years) who had a primary diagnosis (*DX1* of NIS) of AF (i.e., incident AF; *ICD-9 CM* = 427.31) or secondary diagnosis (*DX2–DX25*) of AF (i.e., comorbid or prevalent AF). Identification of a cancer diagnosis concurrent with the primary diagnosis of AF followed prior validated methodology [11]. Metastatic cancer was excluded from this analysis as resource utilization is expected to be positively skewed in this population leading to overestimation. Variables were identified using ICD-9 codes, and other codes included inside NIS are presented in supplemental Table 1. The final cohort selection is presented in supplemental Fig. 1. NIS 2012 to 2015 was utilized for modeling for cost-of-care and length of stay in order to have a contemporaneous analysis.

**Table 1:** Characteristics of hospitalizations, rhythm control procedure utilization, and mortality outcome for primary atrial fibrillation (patient level, financial, and hospital-level) from 2012 to 2015 in cancer vs. non-cancer patients

Variable	Breast cancer (n = 53,800)	*P* value*	Lung cancer (n = 29,535)	*P* value*	Colon cancer (n = 22,525)	*P* value*	Prostate cancer (n = 39,145)	*P* value*	Lymphoma (n = 19,025)	*P* value*	Non-cancer (n = 1,324,335)
Patient characteristics											
Age, years (mean ± SE)	77.4 ± 0.1	< .001	73.5 ± 0.1	< .001	78.3 ± 0.2	< .001	76.8 ± 0.1	< .001	74.0 ± 0.2	< .001	69.5 ± 0.04
Women, %	99.1	< .001	46.5	< .001	56.5	< .001			48.3	< .001	51.6
Race, %		< .001		< .001		< .001		< .001		< .001	
White	87.8		87.0		86.3		85.2		87.3		81.9
Black	6.0		6.8		6.4		8.5		5.6		8.1
Hispanic	3.3		2.9		4.2		3.7		3.8		5.8
Asian or Pacific Islander	1.1		1.4		1.1		0.8		1.3		1.5
Native American	0.2		0.4		0.3		0.2		0.4		0.4
Other	1.5		1.5		1.8		1.6		1.7		2.2
Income quartiles		< .001		.0001		.0001		< .001		< .001	
0–25	23.5		28.8		25.6		23.2		21.5		27.6
26–50	26.0		26.7		28.2		26.2		26.6		26.8
51–75	25.4		24.9		24.2		23.8		25.2		24.3
76–100	25.1		19.6		22.0		26.8		26.8		21.2
Payment source (%)		< .001		< .001		< .001		< .001		< .001	
Medicare	86.1		78.7		86.0		83.6		79.4		65.3
Medicaid	1.6		4.3		1.8		1.0		2.2		5.5
Private	11.1		14.4		10.2		13.0		16.6		23.5
Self-pay	0.5		0.8		0.8		0.5		0.6		3.3
No charge	0.1		0.1		0.1		0.03		0.2		0.4
Others	0.7		1.7		1.2		1.9		1.0		2.1
Comorbidities (%)											
Cardiomyopathy	8.8	< .001	6.6	< .001	8.7	< .001	10.4	< .001	11.8	.0001	11.7
Hypertension	77.7	< .001	69.8	< .001	77.9	< .001	77.6	< .001	70.0	< .001	74.2
Diabetes	26.4	< .001	26.5	< .001	29.4	0.19	27.2	< .001	26.3	< .001	28.8
Obesity	12.5	< .001	8.0	< .001	11.0	< .001	10.5	< .001	10.3	< .001	18.4
Dyslipidemia	50.1	< .001	45.3	< .001	47.6	0.38	56.4	< .001	43.4	< .001	47.6
Coronary artery disease	24.9	< .001	32.9	< .001	32.7	< .001	41.2	< .001	29.5	.12	30.1
Prior myocardial infarction	6.5	.005	9.0	< .001	9.1	< .001	10.2	< .001	7.1	.06	7.4
Prior coronary bypass grafting	4.7	< .001	7.8	.0002	8.4	< .001	12.4	< .001	7.3	.82	7.3
Peripheral vascular disease	6.5	.03	11.5	< .001	8.9	< .001	10.2	< .001	7.4	.80	7.0
Prior TIA/stroke	12.4	< .001	8.5	.79	12.5	< .001	11.8	< .001	9.2	.36	9.7
Chronic renal disease	15.0	.37	14.5	.30	20.5	< .001	22.0	< .001	23.2	< .001	15.3
Chronic lung disease	23.4	.005	56.6	< .001	24.9	.003	23.2	< .001	22.4	.14	22.7
Coagulation disorder	3.5	.26	7.0	< .001	4.8	< .001	5.8	< .001	11.3	< .001	3.3
Smoker	23.4	< .001	57.3	< .001	29.2	.17	36.1	< .001	26.9	.0001	28.0
Total elixhauser’s comorbidities		< .001		< .001		< .001		< .001		< .001	
0	4.7		1.0		3.9		5.4		1.7		8.6
1	17.9		7.3		15.3		18.7		9.2		21.4
2	24.8		18.1		22.6		24.6		19.2		25.4
≥ 3	52.5		73.7		58.2		51.4		70.0		44.6
Elixhauser’s mortality score (mean ± SE)	4.7 ± 0.1	< .001	12.4 ± 0.1	< .001	6.5 ± 0.1	< .001	6.3 ± 0.1	< .001	10.3 ± 0.2	< .001	2.4 ± 0.02
Teaching hospital (%)	52.3	.0001	50.9	.02	50.1	.01	54.0	< .001	56.2	< .001	51.2
Bed size, (%)		.07		.66		.57		< .001		.09	
Small	16.0		16.2		15.9		15.2		15.8		16.7
Medium	28.6		28.1		28.2		28.2		27.4		28.4
Large	55.4		55.7		55.9		56.6		56.8		54.9
Region (%)		< .001		< .001		< .001		< .001		< .001	
Northeast	21.9		20.9		22.5		22.9		23.1		20.7
Midwest	25.5		25.6		26.8		26.0		24.7		24.4
South	37.0		41.0		36.7		36.0		36.4		40.1
West	15.7		12.6		14.1		15.1		15.8		14.8
Rhythm control procedure											
DCCV	10.8	< 0.001	8.2	< 0.001	10.4	< 0.001	13.6	< 0.001	11.4	< 0.001	15.6
AF ablation	3.6	< 0.001	2.5	< 0.001	2.9	< 0.001	4.7	< 0.001	3.7	< 0.001	5.7
Mortality	0.9	0.7	3.2	< 0.001	1.1	0.001	0.8	0.08	2.3	< 0.001	0.9

AF, atrial fibrillation; DCCV, DC cardioversion

*P values presented in the column next to the values for each cancer is versus non-cancer, the values of which are presented in the last column

**Fig. 1:**
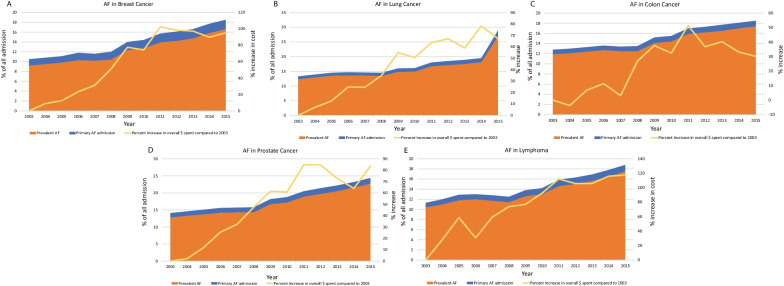
Annual health care costs associated with atrial fibrillation admissions with **A** breast cancer, **B** lung cancer, **C** colon cancer, **D** prostate cancer, and **E** lymphoma. P-trends < 0.001 for the cost of care increase in primary and prevalent AF across all cancers

The outcomes for this study were cost-of-care and length of stay. The cost-of-care or cost of hospitalization was obtained by multiplying HCUP hospital charges with their cost-to-charge ratios [11], and wage index, correcting geographic variations, for a given year. These costs were inflation-adjusted to 2020 using the department of labor charts [11]. Cost-of-care is the estimate US dollar value of the care received by the patient in the hospital as reimbursed by insurance. Length of stay is defined as total number of days spent in the hospital. It was available as a non-computed variable to use in NIS and defined as the subtraction of the admission date from the discharge date. Same-day stays are coded as 0.

Annual variance analysis for NIS datasets was performed using the DOMAIN method for all years. We followed the recommendations from AHRQ for analysis using survey data. Survey‐specific statements with hospital and patient‐level weights were used to obtain national estimates. The Rao–White χ^2^ test was used to compare categorical variables, and a survey‐specific t‐test was used for continuous variables. We used the Cochran–Armitage test of trend for categorical variables. We utilized all cancer and non‐cancer admissions for each year as denominators for comparative annual trends [11]. A survey-specific multivariable linear regression analysis was performed, accounting for multicollinearity and cluster sampled nature of this dataset, to assess the effect of cancer on cost and LOS during primary AF admission, adjusting for age, gender, race, hypertension, diabetes, HCUP mortality score, primary payer as well as all hospital-specific variables (model 1). HCUP mortality score is a score created using a weighted model validated model that includes 29 variables and hence avoids the use of individual covariates and hence prevents multi-collinearity [11]. This model was expanded further by the addition of cardioversion (DCCV), major bleeding during hospitalization or both (models 2, 3, and 4, respectively). The outcomes were verified in those that died during the admission and received an AF ablation as sensitivity analyses. A subgroup analysis was performed for those who received DCCV during admission. All analyses were performed using SAS software, version 9.4 (SAS Institute Inc., Cary, North Carolina).

## Results

Over 13-years, there were 406.5 million weighted admissions in the NIS database of which 51 million (12.6%) had co-morbid cancer. The prevalence of co-morbid AF was 8.2 million (16%) and 35.5 million (10%) among those with vs. those without cancer, respectively (odds ratio = 1.6, 95% confidence interval = 1.5–1.7; *P* < 0.001). Patients with an admission for primary AF with co-morbid cancer were more likely to be older [77.9 vs 75.3 years, standardized difference (SD) = 0.22], male (54.3% vs 48.6%, SD = − 0.12), white (87.6% vs 82.5%, SD = 0.15), and Medicare insured (85.4% vs. 79.7%, SD = 0.15) compared to those without cancer. Prevalence of hypertension (68.8% vs. 69.1%, SD = − 0.008), and stroke (10.5% vs 10.1%, SD = − 0.05) were similar between groups, with less diabetes in patients with cancer (29.0% vs. 32.8%, SD = − 0.08). Cancer-specific characteristics, mortality during primary AF, rhythm control procedure utilization and comparison to non-cancer population in the contemporary year is presented in Table 1.

Over time, both primary and prevalent AF admissions among those with comorbid cancer increased from 1.1% in 2003 to 1.5% in 2015, and 12.3% in 2003 to 21% in 2015, respectively. AF admission rates increased among all study groups (Additional file 1: Table S2). The total estimated inflation-adjusted expenditure on primary AF admissions has increased from $221.3 million in 2003 to $430.3 million in 2015 (a 94.4% increase and P-trend < 0.001) among those with AF and comorbid cancer. In comparison, those with primary AF admission without comorbid cancer saw a 23.9% increase in total cost ($1.6 billion in 2003 vs. $2.0 billion in 2015; P-trend < 0.001). Cost per hospitalization and LOS for AF hospitalization remained higher in recent years in those with comorbid cancer (*P* < 0.001 for both comparisons). Individual cancers showed varying degrees of rise in annual healthcare costs associated with AF hospitalizations, with the highest increase in those with lymphoma (117.5%; Fig. 1A–E).

In patients with a primary AF admission, the cost of hospitalization was lowest (USD 5,767), and LOS was the shortest (1.9 days) among patients without cancer who did not receive DCCV (Table 2). In contrast, cost of care and LOS were significantly higher per hospitalization in those receiving DCCV, when stratified by the presence of absence of comorbid cancer ($7,450 vs. $6,569, and 2.9 days vs. 2.4 days; P-trend < 0.001 for both; Table 2). However, the cost of care was similar in those hospitalizations where the patient underwent ablation for AF or died during the hospitalization regardless of cancer status (Additional file 1: Table S2).

**Table 2:** Length of stay and cost of care among patients with a primary admission for atrial fibrillation

	Length of stay (days)for primary AF hospitalization (2015)	Cost (US dollars) for primary AF hospitalization (2015)
No cancer, no cardioversion	1.9 ± 0.02	5767 ± 238
Cancer, no cardioversion	2.2 ± 0.04	6152 ± 172
No cancer, cardioversion	2.4 ± 0.1	6569 ± 97
Cancer, cardioversion	2.9 ± 0.1	7450 ± 207

In a multivariable regression analysis using data from 2014, cancer was associated with the greater LOS (*P* < 0.001), but not with higher cost of care (*P* = 0.51) for those admitted with AF as a primary cause for admission. Upon controlling for DCCV or major bleeding during the hospitalization in the model, the effect of cancer on LOS remained significant (model above + DCCV: *P* = 0.002; model above + major bleeding: *P* = 0.0005; model above + DCCV + major bleeding: *P* = 0.0002). However, cancer was not associated with LOS or cost of care in subgroups of patients who died or had an AF ablation during the hospitalization (AF ablation subgroup: P-LOS = 0.42, and P-cost of care = 0.08; died subgroup: P-LOS = 0.51, and P-cost of care = 0.71).

**Fig. 2:**
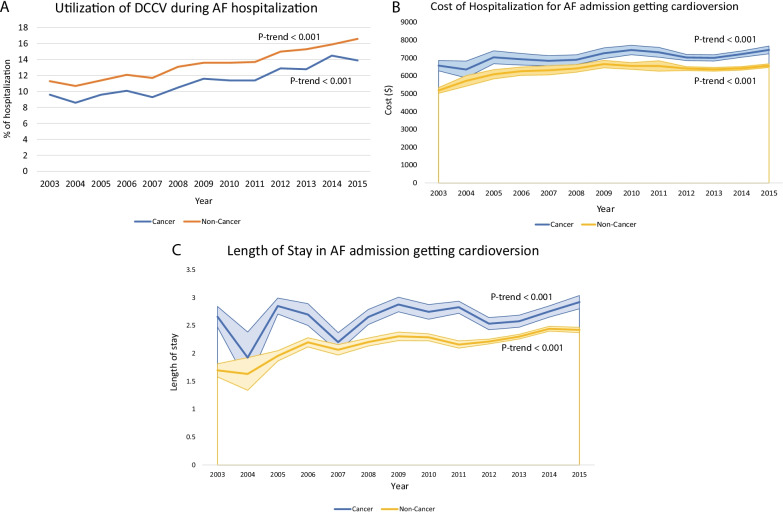
Trends in cardioversion utilization (**A**) and associated cost (**B**) and length of stay (**C**) in cancer and non-cancer patients with admissions for atrial fibrillation. P-trend < 0.001 for utilization, cost, and length of stay

Among the subgroup of patients who underwent DCCV, there has been statistically significant increase in those who get inpatient DCCV in cancer (9.6% in 2003-> 13.9% in 2015) and non-cancer patients (11.3% in 2003- > 16.6% in 2015) (Fig. 2A). This increase in hospitalization for AF with DCCV was accompanied by an increase in LOS and cost of care in both cancer and non-cancer patients (Table 2; Fig. 2B, C). The LOS was higher (2.8 vs 2.4 days) and statistically significant (*P* = 0.05) in a multivariable linear regression analysis (model 1, year = 2014) among those with cancer compared to non-cancer hospitalization for AF that had a DCCV performed. However, there was no difference in cost of care ($7216 vs. $6418; *P* = 0.65) based on cancer as comorbidity in the same multivariable model. Upon considering only those patients with comorbid cancer and primary admission for AF, the group undergoing DCCV had a significantly higher length of stay (2.7 vs. 2.2 days; *P* < 0.001, model 1) and cost of care ($7,093 vs. 6,152; *P* < 0.001, model 1).

## Discussion

This study demonstrates that comorbid AF is more common in patients with cancer that those without, and AF hospitalizations has significantly increased over time, with a higher increase in both the cost of care and LOS among patients with a comorbid diagnosis of cancer. Nevertheless, adjusted analyses demonstrate that only LOS is significantly impacted by a cancer diagnosis. We further evaluated the use of DCCV during primary AF hospitalization. Importantly, there was increased LOS and higher cost of care among patients with cancer who received DCCV for AF compared to non-cancer patients.

Epidemiological trends demonstrate that there is an increasing incidence and prevalence of AF in the general population, with 33.5 million individuals estimated worldwide in 2010 [12]. By 2050, the prevalence of AF in the US is projected to exceed 7.5 million individuals [13]. Epidemiological studies have also demonstrated an association between cancer and AF due to common comorbidities including advanced age, metabolic disorders, or inflammation. Additionally, surgical or medical therapies for cancer may have either direct or indirect effects on the development of arrhythmias [5, 6]. A study of over 15,000 patients found that patients with cancer were more likely to have prevalent AF than those without cancer [6]. Our study confirms these findings with primary and prevalent AF hospitalization significantly increasing in cancer patients between 2003 and 2015 which may be explained by the increasing use of novel cancer therapeutics amongst an aging population.

The management of AF, particularly in those with cancer requires a nuanced approach focusing on rate or rhythm control and thromboembolism prophylaxis due to the potential for unique drug-drug interactions, or treatment disruptions due to bleeding diatheses. Cancer specific algorithms for AF management are ideal but are currently lacking and therefore the same management principles utilized for the general population should generally be applied to cancer patients. Nevertheless, there is increasing evidence of inconsistent application of recommended AF treatment strategies to cancer patients. For example, a recent study demonstrated almost half of cancer patients with an elevated CHADS-VASc score and low HAS-BLED score were not offered anticoagulation for AF [8]. Similarly in a survey of European physicians, 31% preferred low molecular weight heparin or coumadin over a direct oral anticoagulant in cancer patients with AF [14].

While a rate control strategy is preferred for asymptomatic patients or in those whom anticoagulation is contraindicated, for patients with symptomatic AF, including those with heart failure felt to be worsened by AF, a rhythm control strategy is preferable [15]. In fact, based on data from the EAST-AFNET 4 trial, an early rhythm control strategy, even in the absence of symptoms, may lead to fewer long-term adverse events [16]. It should be recognized that cancer patients were not specifically evaluated however. There has been some reluctance to consider DCCV in cancer patients given concerns about long-term efficacy [5]; however, studies in amyloid patients (including patients with the malignant variant AL amyloid) have shown high rates of procedural success, similar to non-amyloid patients ^17^. Moreover, among patients with comorbid cancer who underwent DCCV, both LOS and cost of care was greater than non-cancer patients. Various factors could explain this finding including a reluctance to quickly offer DCCV to cancer patients due to above mentioned biases leader to longer and more expensive hospitalization. Alternatively, patients with AF and a comorbid cancer diagnosis may have more medical issues requiring upfront management prior to DCCV compared to patients without cancer. Cancer diagnosis was not associated with a difference in LOS or cost of care for patients receiving inpatient AF ablation procedure. It should be recognized however that this procedure is generally performed only on clinically stable patients without other serious comorbidities.

There are several limitations of our study that warrant consideration. Because of the reliance on *ICD-9-CM* codes, we were unable to determine the difference between recurrent and new-onset AF, and the physician-perceived indication for hospital admission by specific cancer type. Patients with a diagnosis of metastatic cancer were excluded from our analyses; while this may impact overall health care utilization data, we felt physician perceptions about this subset of patients would adversely bias our results. We could not determine the percentage of patients with active cancer compared to a previous history of the disease (i.e. cancer survivors), nor the duration of a particular cancer diagnosis. We were also unable to determine specific cancer treatments and whether those therapies had any impact on AF hospitalization trends. Additionally, administrative data do not account for unmeasured factors like patient care preference, physician perception of prognosis, and shared decision making on the delivery of care.

## Conclusion

AF related admissions are increasing for all populations especially amongst those patients with a comorbid diagnosis of cancer, including all cancer subtypes evaluated. Among those patients who underwent DCCV, cancer patients had longer length of stay and increased health care costs. Further research into the factors related to AF mechanism and presentation in cancer patients is necessary, which may ultimately help improve health care cost and utilization.

## Supplementary Information

**Additional file 1.** Supplementary methods, figures and tables.

## Data Availability

The data underlying this article were provided by HCUP under license. Data will be shared on request to the corresponding author with the permission of HCUP.

## Abbreviations

AF: Atrial fibrillation
AHRQ: Agency for Healthcare Research and Quality
DCCV: Direct current cardioversion
HCUP: Healthcare Cost and Utilization Project
LOS: Length of stay
NIS: National Inpatient Sample
