# Supplementary material for: Length of stay and cost of care associated with admissions for atrial fibrillation among patients with cancer
Source: BMC Cardiovasc Disord. 2022 Jun 17;22:272. doi: 10.1186/s12872-022-02697-4 (PMC9205123; doi:10.1186/s12872-022-02697-4)
Supplement: Supplementary file 1 — Additional file 1. Supplementary methods, figures and tables. [file 12872_2022_2697_MOESM1_ESM.docx]

**Supplementary Methods**

**National Inpatient Sample**

The National Inpatient Sample (NIS) is an inpatient database in the US^1^ developed by the Agency for Healthcare Research and Quality (AHRQ). It is comprised of a 20% nationwide sample of all inpatient discharges from US hospitals, excluding patients admitted for observation status, short-term rehabilitation hospitals, long-term non–acute care hospitals, psychiatric hospitals, and alcohol or chemical dependency units. This dataset contains de-identified information regarding each hospitalization, including demographic characteristics, co-morbidities, discharge diagnoses, procedures, outcomes, and total cost of the admission. The design of the NIS changed twice during the study^2^. Between 2003 and 2011, the NIS comprised all inpatient discharges from a 20% nationwide random sample of acute-care hospitals in the US. However, in 2012, instead of including all discharges from the 20% nationwide sample, the database was constructed using a systematic sampling of 20% of discharges from the hospitals stratified by hospital, census division, ownership status, location, teaching status, and bed size, as well as patient diagnosis-related group and admission month. To facilitate patient-level trend analysis, a new set of weights called “trendwt” was developed for data from previous years (1993-2011)^1, 3^. The trend weights replaced the original NIS discharge weights for trend. We used trend weights for all patient and hospital level analyses^3^. In 2015, NIS moved to the International Classification of Diseases, Tenth Revision, Clinical Modification (ICD-10-CM) format of data collection, and hence, data was available only through September 2015^4^. Data collection methodology was unchanged compared to 2012.

**Supplemental Figure1** Flow chart showing methods

**Supplemental table 1**: Diagnosis codes used in the study

| **Procedure** | **ICD-9 code** | **CCS code** | **Comorbidity field from NIS** |
| --- | --- | --- | --- |
| **Cohort Creation** |  |  |  |
| Cancer | 162.xx,174.xx,175.xx,153.xx,154.xx,185.xx,182.xx,183.xx,188.xx,189.xx,200.xx,201.xx,202.xx,204.xx,205.xx,206.xx,207.xx,208.xx,155.xx,156.xx,172.xx,193.xx,157.xx | 11,12,13,14,15,16,17,18,19,20,21,22,23,24,25,26,27,28,29,30,31,32,33,34,35,36,37,38,39,40,41,42,43,44,45 | CM_LYMPH,CM_TUMOR* |
| Breast Cancer | 174.xx,175.xx | 24 |  |
| Lung Cancer | 162.xx | 19 |  |
| Colon Cancer | 153.xx,154.xx | 14 |  |
| Prostate Cancer | 185.xx | 29 |  |
| Lymphoma | 200.xx,201.xx,202.xx | 37,38 | CM_LYMPH |
| **Comorbities/co-diagnosis^‡^** |  |  |  |
| Cardiomyopathy | 425.xx |  |  |
| Known Coronary Artery Disease | 414.00, 414.01, 414.02, 414.03, 414.04, 414.05, 414.06, 414.07 |  |  |
| Prior Myocardial Infarction | 412.xx |  |  |
| Prior Coronary Bypass Grafting | V45.81 |  |  |
| Prior TIA/Stroke | 438.xx,V12.54 |  |  |
| Atrial Fibrillation | 427.31 |  |  |
| Hypertension |  | 98,99 | CM_HTN_C |
| Diabetes |  | 49,50 | CM_DX,CM_DMCX |
| Obesity | 278.xx |  | CM_OBESE |
| Chronic Kidney Disease |  | 158 | CM_RENLFAIL |
| Chronic Lung Disease |  |  | CM_CHRNLUNG |
| Dyslipidemia |  | 53 |  |
| Peripheral Vascular Disease |  | 114 | CM_PERIVASC |
| Smoking | 305.1x, V158.2 |  |  |
| Coagulation disorder |  |  | CM_COAG |
| **Procedures** |  |  |  |
| Ablation | 37.34,37.33 |  |  |
| DC- cardioversion (DCCV) | 99.61,99.62 |  |  |

**Supplemental Table 2:** Outcomes associated with atrial fibrillation among cancer patients.

|  | **Non-Cancer** | **Cancer** | **Breast Cancer** | **Lung Cancer** | **Colon Cancer** | **Prostate Cancer** | **Lymphoma** |
| --- | --- | --- | --- | --- | --- | --- | --- |
| **Prevalence of AF in 2003 (%)** | 8.7 | 12.3 | 10.5 | 13.3 | 12.8 | 14.1 | 11.3 |
| **Prevalence of AF in 2015 (%)** | 12.4 | 21 | 18.5 | 28.9 | 18.5 | 24.4 | 18.8 |
| **Primary AF admission in 2003 (%)** | 1.1 | 1.1 | 1.3 | 1 | 0.9 | 1.3 | 0.9 |
| **Primary AF admission in 2015 (%)** | 1.3 | 1.5 | 1.9 | 2 | 1.1 | 1.7 | 1.3 |
| **Cost per hospitalization for primary AF in 2003 ($±SE)** | 5,518±107 | 6,079±146 | 5,815±175 | 6,728±227 | 6,194±232 | 5,797±200 | 6,662±278 |
| **Cost per hospitalization for primary AF in 2015 ($±SE)** | 5,850±63 | 6,360±82 | 6,032±115 | 6,947±162 | 6,230±0.1 | 6,519±151 | 6,876±239 |
| **Relative cost of care over time 2015 compared to 2003 (total health care dollars spent annually) (%)** | 23.9 | 94.4 | 95.1 | 67.2 | 30.2 | 83.7 | 117.5 |
| **Cost per hospitalization for primary AF in 2015 for those who had cardioversion ($±SE)** | 6,569±97 | 7,450±207 | 7,160±300 | 8,719±587 | 7,568±501 | 6,724±287 | 9,404±713 |
| **Cost per hospitalization for primary AF in 2015 for those who had ablation ($±SE)** | 25,039±629 | 24,588±913 | 23,096±1,670 | 23,976±2,115 | 25,836±2,219 | 23,826±1,289 | 31,345±2,709 |
| **Cost per hospitalization for primary AF in 2015 for those who died during the hospitalization ($±SE)** | 10,136±571 | 10,244±651 | 7,812±432 | 12,641±666 |  | 10,087±956 | 10,921±400 |
| **Length of Stay for primary AF hospitalization in 2003 (days±SE)** | 2.0±0.1 | 2.5±0.1 | 2.6±0.1 | 3.1±0.1 | 2.6±0.1 | 2.1±0.1 | 2.6±0.2 |
| **Length of Stay for primary AF hospitalization in 2015 (days±SE)** | 2.0±0.1 | 2.4±0.1 | 2.4±0.1 | 2.9±0.1 | 2.4±0.2 | 2.3±0.1 | 2.6±0.2 |
| **Length of Stay for primary AF hospitalization in 2015 for those who had cardioversion (days±SE)** | 2.4±0.1 | 2.9±0.1 | 2.8±0.3 | 3.1±0.5 | 3.4±0.4 | 2.7±0.2 | 3.1±0.5 |
| **Length of Stay for primary AF hospitalization in 2015 for those who had ablation (days±SE)** | 2.3±0.1 | 3.0±0.3 | 3.1±0.2 | 3.4±1.4 | 3.7±0.6 | 3.0±0.3 | 3.2±0.4 |
| **Length of Stay for primary AF hospitalization in 2015 for those who died during hospitalization (days±SE)** | 2.1±0.3 | 2.7±0.5 | 1.2±0.1 | 5.3±0.3 |  | 4.4±0.3 | 3.4±0.4 |

^Black shading represents clusters with < 10 observations and not reported per HCUP data use agreement. Abbreviations: AF: atrial fibrillation, $: united states dollar cost, which is inflation, geography adjusted, SE: standard error^

**Reference**

1. Overview of the National (Nationwide) Inpatient Sample (NIS). 2018;2018.

2. Houchens RL, Ross DN, Elixhauser A and Jiang J. Nationwide Inpatient Sample Redesign: Final Report. 2014;2018.

3. Marzolini S, Blanchard C, Alter DA, Grace SL and Oh PI. Delays in Referral and Enrolment Are Associated With Mitigated Benefits of Cardiac Rehabilitation After Coronary Artery Bypass Surgery. *Circ Cardiovasc Qual Outcomes*. 2015;8:608-20.

4. Barrett ML. 2015 Healthcare Cost and Utilization Project (HCUP) National Inpatient Sample: Change in Structure and Data Elements Caused by Transition to ICD-10-CM/PCS. 2017;2018.
